# Integrated BIM and VR to implement IPD mode in transportation infrastructure projects: System design and case application

**DOI:** 10.1371/journal.pone.0259046

**Published:** 2021-11-11

**Authors:** Ziqi Hao, Wensheng Zhang, Yunche Zhao

**Affiliations:** 1 School of Traffic and Transportation, Shijiazhuang Tiedao University, Shijiazhuang, China; 2 Science & Technology Research & Development Center, Shandong Provincial Communications Planning and Design Institute Group Co., Ltd, Jinan, China; Al Mansour University College-Baghdad-Iraq, IRAQ

## Abstract

The complex design of transportation infrastructure hinders communication between different roles in the project, which makes it difficult to promote the Integrated Project Delivery (IPD) mode. This paper discusses a design simulation and communication system based on Building Information Modeling and Virtual Reality for transportation infrastructure (DSC-BV-TI system), integrated with BIM, with VR developed by using a game engine. Based on an analysis of the user’s demand, the system introduces a three-dimensional BIM model of traffic infrastructure in an immersive VR environment and realizes the simulation design, weather simulation, virtual driving, sight distance calculation, visual simulation and other functions of traffic infrastructure project by using the system’s safety assessment and scheme decision. The system is applied to the design of the Jinjiazhuang Extra-Long Tunnel project of the Yan-Chong Expressway in Hebei Province, which was built for the 2022 Winter Olympics. The results show that, using the DSC-BV-TI system, the designer has completed a display of the overall scheme: the user can use the steering wheel to drive a vehicle; use the head-mounted display to play the picture; realize the simulation and interaction in a variety of simulated weather conditions and environments; and use IPD mode to communicate and make decisions on the design scheme of the traffic infrastructure, tunnel speed limit and other aspects that play a key role. The DSC-BV-TI system has 8 advantages and 4 disadvantages identified through a questionnaire survey, the advantages including high fidelity, high efficiency and low cost. At the same time, according to the research results, three suggestions to help improve the system are discussed. DSC-BV-TI system as a communication bridge between the design team and other stakeholders reduces the communication gap and promotes the implementation of the IPD mode in transportation infrastructure projects.

## 1 Introduction

With the continuous expansion of the world’s population and the increasing diversification of transportation needs, transportation infrastructure projects have become more and more complex and challenging [1–3], which also makes greater demands on the knowledge of project-related executives [4, 5]. A large-scale transportation infrastructure project is very complicated. During its design process, the design team, the contractor, the government department and other project stakeholders need to communicate regularly to ensure that the final design results can meet the needs [6, 7]. Whether information can be accurately communicated in this process is a key factor in reducing misunderstandings, improving communication efficiency and speeding up the design process [8, 9].

The Integrated Project Delivery (IPD) model is defined by the American Institute of Architects. Its concept relies on multidisciplinary and team collaboration, encouraging all stakeholders to participate in the design, construction and management of the project, so as to avoid potential conflicts of interest and project disputes [10, 11]. During the development of a transportation infrastructure design project, designers, investors, the government and even the public need to have a clear understanding of the design plan, and work together to ensure that the construction of transportation infrastructure can meet the requirements [12]. However, the participants are currently limited by the traditional mode, and the sense of collaboration is weak; the advantages of the IPD mode cannot be fully utilized and it is difficult to carry out mutual cooperation among multi-party teams, and to maximize the value of project productivity [13, 14]. Therefore, it is necessary to provide a communication platform for designers, inspectors and stakeholders in the design process of transportation infrastructure projects to promote communication.

In recent years, the advent of Building Information Modeling (BIM) has extended the design scheme to three-dimensional space [15, 16]. BIM brings a new expression paradigm to the entire life cycle of the project to manage the design data in digital format generated in the project [17, 18]. Most of the BIM used in transportation infrastructure projects should be classified as Horizontal BIM [19–21]. The IPD mode is based on the integration of information and knowledge. This form of organization and management requires the collaborative work of all parties involved. The highly integrated BIM engineering information makes it naturally the main technical support under the IPD mode and the application of BIM in the IPD mode is widely regarded as one of the best uses of BIM [2, 22, 23].

However, for non-professionals, the 3D model of BIM is still too complicated and offers a poor interactive experience, which cannot well promote collaboration between teams [24, 25]. Virtual Reality (VR), as a multimedia technology that provides an immersive experience, allows users to interact with digital objects in a virtual environment in real time, which can enhance communication and help integrate discussion results. Carrying out the evaluation work in the VR environment helps users to better understand the project and simplifies the communication process, which can bring many benefits for the virtual design, prototyping and simulation of transportation infrastructure [26–28].

In the design stage of transportation infrastructure, there are few studies that combine BIM and VR technology to develop a simulation and communication platform that can be applied to the IPD mode. The main purpose of this research is to enhance the stakeholders’ understanding of the project, thereby simplifying the communication process between all parties, and optimizing the design results. To this end, a design simulation and communication system based on BIM and VR for transportation infrastructure (DSC-BV-TI system) was developed, which uses a game engine to integrate BIM and VR technology, allowing designers, investors and other stakeholders to participate in the discussion of transportation infrastructure in the pre-design stage.

An important control project is the Yan-Chong (Beijing Yanqing-Zhangjiakou Chongli) Expressway constructed for the 2022 Winter Olympics: it includes the Jinjiazhuang Extra-Long Tunnel, which has been certified by the Guinness Book of World Records as the world’s longest spiral highway tunnel.

This research is based on the IPD collaborative design concept, and on the superiority of BIM and VR in transportation infrastructure projects. The DSC-BV-TI system has been designed and developed using the Unity game engine, which realizes the functions of simulation design, weather simulation, virtual driving, line-of-sight calculation and safety assessment as the main platform for the realization of the IPD mode in the design of transportation infrastructure projects. The DSC-BV-TI system has been practically applied in the Jinjiazhuang Extra-Long Tunnel project of the Yan-Chong Expressway in Hebei Province to verify its effectiveness in optimizing design schemes and results. The system provides a communication platform for the design team, acceptance experts and other stakeholders. Users use the HTC Vive head-mounted display and Logitech G29 steering wheel to roam and browse in the virtual simulation scene, and feedback their opinions and improvement suggestions to the designer for modification, so as to find and solve problems in advance.

## 2 Literature review

### 2.1 Application of BIM in transportation infrastructure project

BIM has been widely used in the construction industry, and its mature methods and technologies have shown great potential for benefiting the transportation industry; there have been numerous applications involving BIM in the transportation infrastructure field in recent years. Wang et al. applied BIM technology to the delivery management of underground rail transit projects, providing technical support for later operation and maintenance [29]. Liu et al. proposed a BIM-based design and construction improvement scheme in a large-span steel box arch bridge project, which verified the potential of BIM for improving bridge design and construction [30]. Park et al. integrated meshless analysis methods into Industry Foundation Classes (IFC) to solve the problem of inefficient meshing of bridge BIM models for finite element analysis [31].

Tang et al. built a 3D road model with controllable pavement parameters and supported the analysis and calculation of pavement structure by relying on the fast-modeling advantage of Dynamo [32]. Kim et al. proposed an object-oriented data model for automatically analyzing the cost and time required for different road alignment options and identifying the best alignment design option [33]. Wang et al. validated the feasibility of applying BIM technology in a subway project using IPD [34]. BIM is considered to play an important role in the design process of shield tunnels, and the 3D model based on the IFC standard can serve for volume statistics and cost calculation in the future [35]. The application of BIM in the design stage of mountain tunnel projects to create multi-level-of-detail (LOD) 3D models, for model visualization and schedule simulation, can provide considerable cost savings for the project [36]. In addition, several studies have been devoted to the design and construction of transportation facilities, such as airports and railroads [19, 37]. However, for the application of IPD mode in a transportation infrastructure project, the aforementioned research still has certain shortcomings: Although research on BIM in the field of transportation infrastructure covers various aspects, such as bridges, highways, and tunnels, but most of the research focuses mainly on the discussion of 3D modeling methods, structural analysis, and volume statistics of the facilities, etc.; there is a lack of methods to verify the design results of transportation infrastructure projects in the early design stage. The problem we wish to address is how to take advantage of BIM in the design stage of transportation infrastructure to involve all stakeholders, discuss and improve the design results, and then achieve the optimal design solution.

### 2.2 Promoting communication through IPD mode in a transportation infrastructure design project

In the transportation infrastructure field, using BIM and other multi-source heterogeneous data to build a multi-participant platform, most studies on enhancing IPD mode promotion have certain similarities. Specifically, they involve constructing BIM models of highways, tunnels or bridges, and using drone tilt photography to collect GIS spatial data, such as terrain and images, and then developing comprehensive 2D/3D integrated monitoring on the web or desktop, to present the user with real geographical location information and integrate three-dimensional visual expression. This is one of the most widely used ways of combining BIM and GIS [38–42]. In these studies, the BIM and GIS data of the expressway are mainly used to visualize the transportation infrastructure and manage the model data. However, relying only on observation and monitoring, the experience of the road design results is still one-sided, unless it is an obvious design error; otherwise, this method is not sufficient to support optimization of the design results.

Unlike other projects, the important feature of a transportation infrastructure project is that one of its main functions is to support the operation of vehicles [43]. Therefore, the subjective perception and evaluation of highway driving from the driver’s view is an important basis for measuring engineering design results, and this feature is one of the key entry points for the application of the IPD mode in the transportation infrastructure field. A 3D scene constructed in virtual space using BIM and GIS data is typically highly consistent with the real scene [44]. Therefore, using this environment for virtual driving simulation can easily gather the driver’s forward-looking perception, feedback and evaluation of the engineering design results, and then improve and optimize these results, which is a sound basis for the promotion of the IPD mode in the transportation infrastructure project [45]. However, to perform virtual driving simulation, it is not enough to rely on only 3D models. Driving scenes should also include natural phenomena, such as light, weather and time. Whether the system can provide users an immersive driving experience is also a key question worthy of attention.

### 2.3 Advantages of applying immersive Virtual Reality technology in engineering design

Immersive VR technology enables users to experience project design effects at an early stage, and practitioners can use it to evaluate engineering results, design options and material selection, and coordinate the construction plan to improve design plans in the early stages of a project [46]. Game engines can simulate realistic physical feedback and light effects, and, combined with VR technology, they can create realistic virtual reality simulation scenarios and interactive experience environments in which to enhance the performance of 3D models [47]. VR technologies based on game engines are considered effective tools to immerse non-expert users and engage them in the design discussion process [48–50]. Moreover, VR can enhance the cognitive and communication effects of a project through real-time interaction in the early stage of design. Wei and Li et al. adopted VR technology in interior space design and landscape design and concluded that it can better communicate design intent compared to traditional methods and help broaden design ideas, as well as identify problems in advance [51–53].

These emerging technologies, which have been well applied in other fields, are demonstrations of the potential for application of IPD mode in a transportation infrastructure project.

Previously, there were few studies using virtual driving and other simulation technologies to simulate and verify the design results of transportation infrastructure. Thanks to the improvement of software ecology and the innovation of hardware equipment, the difficulty and cost of VR research and development have been greatly reduced, making it possible to simulate and optimize transportation infrastructure in the design stage of a project.

In recent years, many projects have used virtual reality technology for visualization, analysis, and evaluation in the design process, but most of them focus on buildings in the form of vertical structures, while the engineering experience of building facilities in the form of horizontal structures differs significantly from vertical structures in terms of modeling and requirements, and so the lessons learned cannot be fully applied to transportation infrastructure. When aiming to enhance the communication effect in the pre-design stage of transportation infrastructure projects, it is still necessary to study the application method and realization effect of VR. Therefore, research related to BIM and VR in transportation infrastructure projects has broad prospects for development.

## 3 System architecture design and development

### 3.1 IPD mode implementation framework

**Fig 1:**
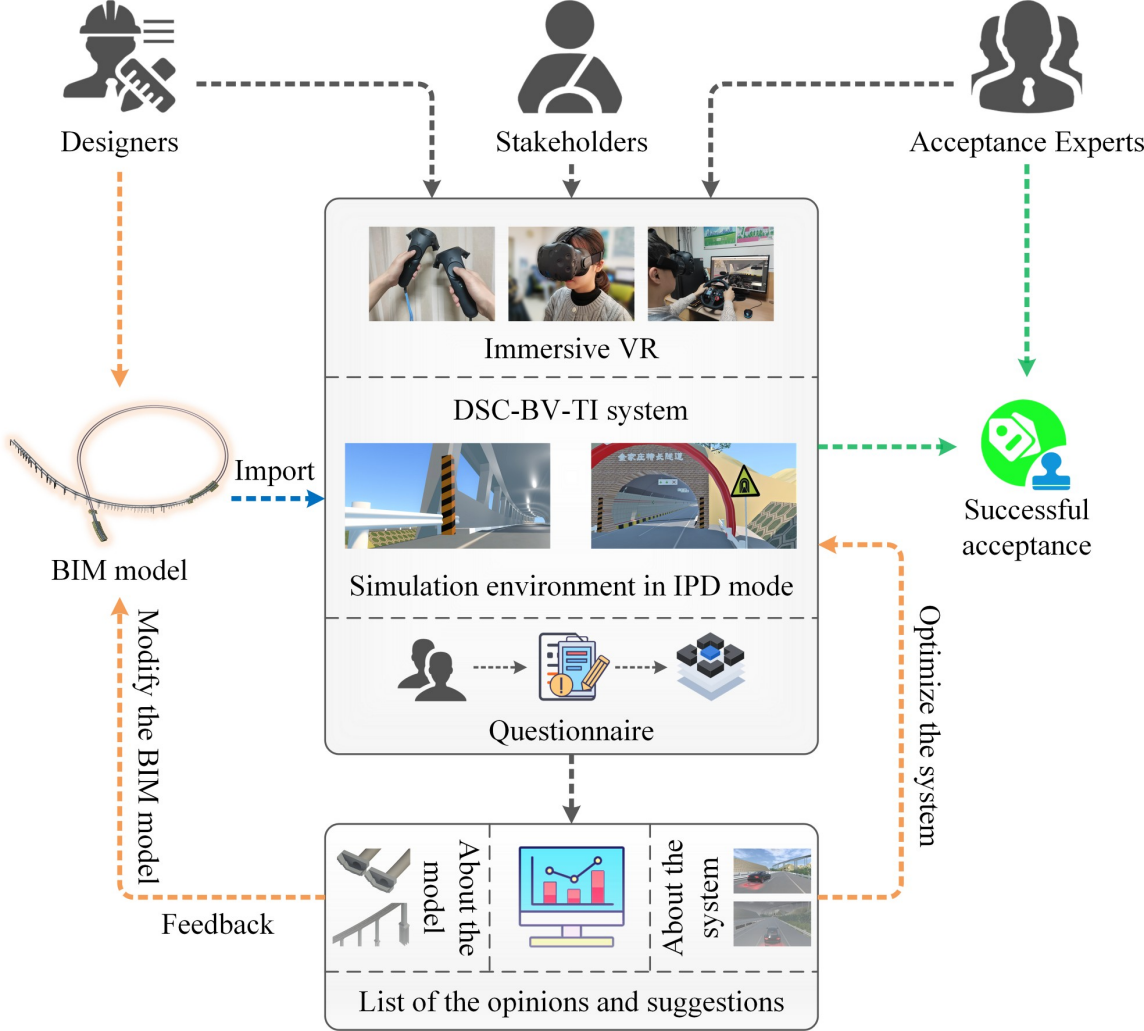
Implementation framework of IPD mode.

Fig 1 shows the IPD mode implementation framework of the DSC-BV-TI system. In this study, the main participants include designers, acceptance experts and stakeholders (e.g., government personnel and the public). All participants use virtual reality equipment to get an immersive VR experience in the DSC-BV-TI system. In the DSC-BV-TI system, the BIM model constructed by the designer is integrated, and the real terrain scene and physical feedback are established. The users can experience the design results of the project through virtual simulation and provide their opinions. By collecting the results of the questionnaire survey, all the review opinions and revision suggestions can be summarized and counted. After receiving the feedback, the designer will modify and optimize the BIM model of the roads and bridges in the transportation infrastructure project and update it in the DSC-BV-TI system, until the design results pass the review of the acceptance expert. The following sections describe the hardware equipment, software architecture design and development process of the DSC-BV-TI system.

### 3.2 Hardware equipment

The technical specifications of the hardware equipment required to study the engineering design scheme in the immersive VR environment provided by the DSC-BV-TI system are listed in Table 1.

**Table 1:** Technical specifications of hardware equipment required for immersive VR simulation.

Tool	Technical specification
Desktop PC	• CPU: Intel^®^ Core™ i9-9900KF @3.60GHz (8 Core) (@5.00GHz Intel^®^ Turbo boost)
	• Graphics card: NVIDIA AORUS GeForce^®^ RTX 2060 Super™ with 8GB GDDR6 VRAM (HDMI 1.4 Output)
	• Display: 23.8″ (16:9) Quad HD (2560×1440) 60Hz
	• Memory: HDD (512GB SATA3 SSD + 3TB 7200RPM SATA HDD); RAM (64GB DDR4)
	• OS: Microsoft Windows 10 Home (64bit)
Logitech G29	• Wheel: 900° Rotation; Hall-effect steering sensor; Dual-Motor Force Feedback
	• Pedals: Nonlinear brake pedal; Self-calibrating
	• Materials: Wheel cover (Leather); Wheel spokes (Anodized aluminum); Steering shaft, Shifter paddles (Brushed stainless steel)
HTC Vive	• Screen: 3.6″ Double AMOLED 90Hz
	• Resolution: Combined 2160×1200
	• Field of view: 110°
	• Sensors: Steam VR tracking technology; G-sensor correction; Gyroscope; proximity
	• Control handle: Multi-function touch panel; Grip button; Two-stage trigger; System button; Menu button
	• Input: HDMI, USB 2.0; 3.5 mm headset; Bluetooth

### 3.3 The overall architecture design of the system

By communicating with the design team, the contractors and the community, we identified the transportation infrastructure demand for the design, simulation and communication in the design cycle. After analyzing the functions provided by the existing commercial software, the functions of the DSC-BV-TI system were customized. Furthermore, confirmation was obtained and adjustments made with the aforementioned stakeholders, and finally the functional composition of the system was determined.

Based on the requirements of the design stage, a simulation scene was created. First, the initial BIM model of the transportation infrastructure was developed using Autodesk Revit and the BIM model conversion method based on element ID was studied. After the lightweight BIM model was created, it was converted into a format supported by the game engine for simulation in the VR environment. Finally, materials were added, lighting adjusted, rendering and baking techniques established in the game engine to build a high-fidelity scene environment.

**Fig 2:**
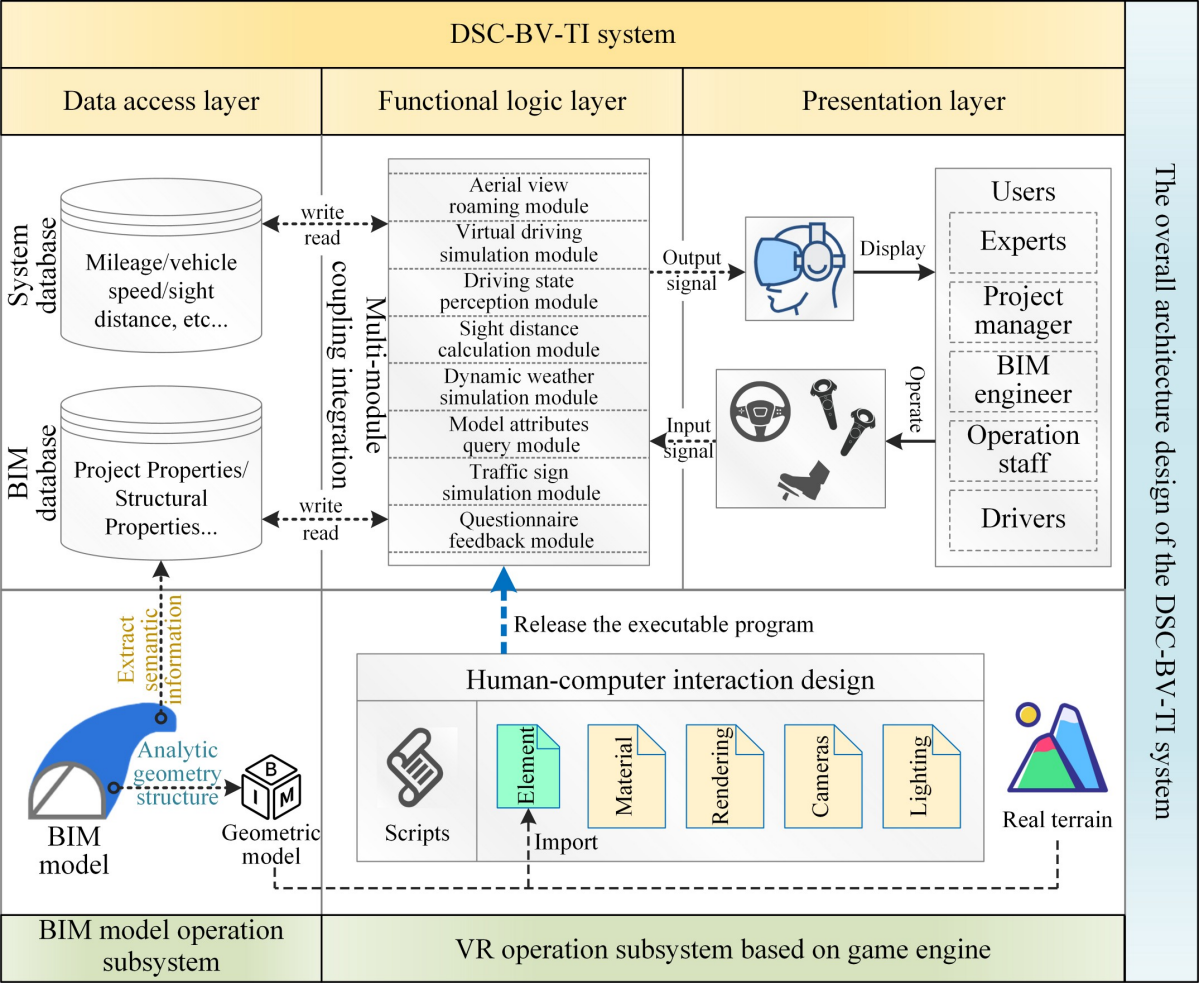
The overall architecture design.

Fig 2 shows the overall architecture design of the DSC-BV-TI system. The system includes a main system and two subsystems. The architecture of the main system consists of three layers: the data access layer, the functional logic layer, and the presentation layer. The data access layer is responsible for storing the data generated by the system, and updating the attribute information of the BIM model from the database. The functional logic layer is the core part of the system. The functional modules developed by the game engine are coupled in this layer, which reads data from the data access layer, and realizes human-computer interaction with users in the presentation layer. At the presentation layer, visual signals such as the virtual reality environment and function realization effects are presented to the user through the screen of HTC Vive. The users operate the system by operating the steering wheel, the brake pedal and the HTC Vive control handle after seeing the played screen.

The database in the system is subdivided into two parts: system database and BIM database. The system database is responsible for storing operation and maintenance data, such as mileage, vehicle speed, and line of sight generated by the DSC-BV-TI system during operation. The BIM database is connected with the BIM software, and is responsible for storing and synchronously updating the engineering attribute and structural attribute information of the transportation infrastructure BIM model. It is mainly used to query the component details of the model in the DSC-BV-TI system and check the design results.

In order to enable the operation of the DSC-BV-TI system, there are two proposed subsystems as support in the research; namely, the BIM model operation subsystem, and the VR operation subsystem based on the game engine, as shown in Fig 2. The design and development process of each subsystem are described in detail as in the next sections.

#### 3.3.1 BIM model operation subsystem

At this stage, the semantic information of the BIM model is extracted and stored in the BIM database through the BIM model operation subsystem. Following this, in the VR platform, the semantic information is associated with the geometric model based on the element ID. Therefore, the semantic information stored in the BIM database will be updated with the status of the BIM model components and synchronized with the DSC-BV-TI system.

**Fig 3:**
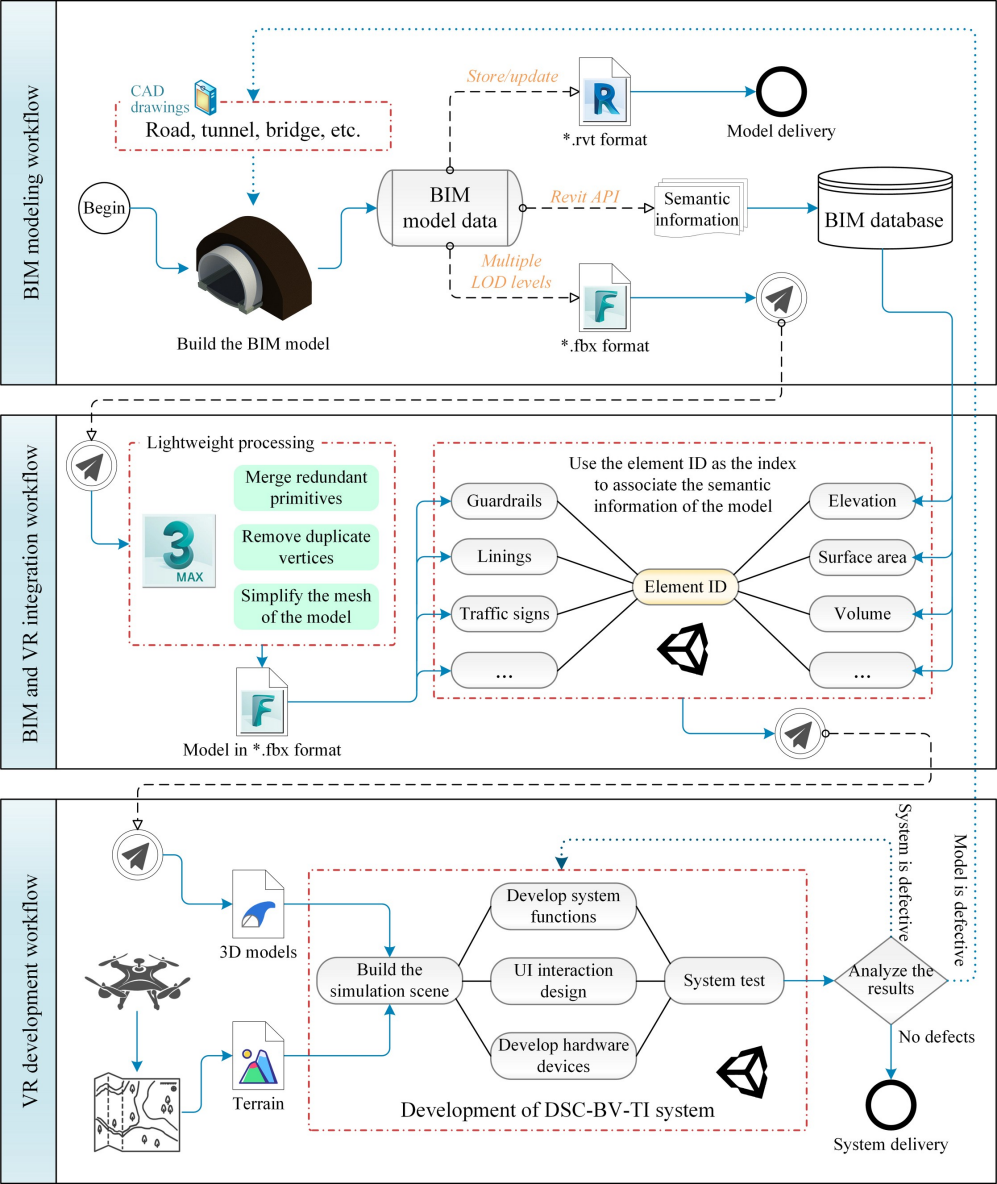
Flowchart of BIM and VR integration.

The first section, BIM modeling workflow, in Fig 3 describes the main process of BIM modeling in the DSC-BV-TI system. In the BIM model operation subsystem, the designers build BIM models of transportation infrastructure, such as roads, tunnels and bridges, and provide BIM model engineering files in *.rvt format to the project acceptance team. Simultaneously, in order to realize the integration of BIM and VR, it is necessary to export BIM models of different LOD levels from Revit in *.fbx format, and decompose the BIM model into the smallest component units, traverse to read their semantic information, use the element ID (the ID is unique in the current model) as an index, and store it in the model database through the export function provided by the Revit API.

Although the 3D model in the *.fbx format is relatively simplified compared with the *.rvt format, there are still a large number of BIM components included in the overall transportation infrastructure project. Some components are small in size, but they have a complex structure and contain many irregular surfaces and curves, which means that a lot of processing work is required in the game engine. Therefore, the models needs to be further simplified by Autodesk 3ds Max. In 3ds Max, there are three ways to make the 3D model more lightweight: 1) Merge primitives of the same material and the same family type, such as merging hundreds of the same tunnel guide lights into a single object; 2) Inspect small components with complex structure and remove isolated vertices; 3) For some components with more irregular surfaces, reduce the number of meshes. The purpose of this step is to optimize the model so that it can be displayed smoothly with high performance quality in the VR environment.

The 3D model in *.fbx format does not carry the semantic information of the model, but the semantic information is very important for the design work. In order to solve this problem, when importing the model into the game engine, the element ID is used as an index to associate the geometric model of the component with its corresponding semantic information through the BIM database. Based on this method, the structural attributes and engineering attribute information of components can be easily queried in the VR environment.

#### 3.3.2 VR operation subsystem based on game engine

Import the simplified BIM model into the game engine, and create a VR roaming scene by rendering the environment. As the development platform of the DSC-BV-TI system, the game engine is a key tool for the realization of interactive functions and real-time rendering of graphics. In this study, Unity was chosen as the game engine, mainly because Unity has obvious advantages in cross-platform compatibility and adaptability, and has abundant plug-in resources. Using the game engine to develop the system allows convenient manipulation of the physical properties, lighting, and materials of the elements.

Importing the BIM model into the game engine and building the simulation scene includes the following three steps:

***Step 1***: Build a VR roaming simulation scene. First, capture high-resolution ground images of the real environment based on drone tilt photography to create the 3D real scene model. Then, import the model into the game engine and convert it to terrain format. Finally, add plants, rocks, water and other elements to the terrain to complete the environment settings for the scene.

***Step 2***: Import the BIM model of the transportation infrastructure. The BIM model contains a large number of components. In order to ensure that the spatial topological relationship within the BIM model is correct after importing the game engine, when using 3ds Max to optimize the model, it is necessary to ensure that the base point of each component remains unchanged. After the model is imported into the game engine, the original texture and semantic information will be lost. Therefore, based on the element ID value carried by each component, the corresponding information should be read and associated from the BIM database.

***Step 3***: Realize physical feedback simulation. Add Mesh Collider and Rigidbody component to BIM models such as roads, bridges, tunnels, etc. These two are the core components for realizing physical effects like collision feedback and free fall in the game engine. In addition, realize lighting simulation in the scene with two kinds of lighting elements: sunlight and artificial light. During development, attention should be paid to parameter settings, such as illumination angle, distance and position, and cameras should be added to create a VR scene.

VR scenes are specially used to provide an immersive experience environment. The interactive mode and interface design in the simulation scene is the key to enhancing the user’s virtual experience and the degree of realism of the scene, such as weather, fallen leaves, natural wind, etc. After this, it is necessary to develop related functional modules to complete the VR simulation system.

### 3.4 System function module design

The DSC-BV-TI system integrates BIM and VR technologies, allowing users to simulate driving, human-computer interaction and convey design intent in a BIM+VR environment. The DSC-BV-TI system includes eight main modules: aerial view roaming module; virtual driving simulation module; driving state perception module; sight distance inspection module; dynamic weather simulation module; model attribute query module; traffic sign simulation module; and questionnaire feedback module. The detailed description of each module of the system is as follows:

- Aerial view roaming module

Users can experience the whole project via the aerial view roaming module to review the design results of the project. They can roam to the point of interest (POI) in the project in the VR environment to view the graphic introduction. In the immersive experience, users can check whether the design results of the project meet the requirements, based on professional knowledge, experience and simulation.

- Virtual driving simulation module

This module realizes the driving simulation of the vehicle based on the vehicle dynamics. The system provides three ways to control the vehicle: Vive handle, keyboard or steering wheel kit. Correspondingly, users can choose to directly use the computer screen (suitable for users who may suffer from VR motion sickness) or use a head-mounted display (which can provide an immersive experience) to obtain visual information. Users can drive various vehicles on the road to be built, experience and discuss the design result of the road (e.g., line shape, lane width, traffic signs, etc.) via the system’s functions.

- Driving state perception module

This module can gather real-time speed, viewpoint height, current station number, mileage and other data, and store it based on time series to record the displacement process of the vehicle. By cooperating with other modules, simulation data collection under complex environmental conditions can be completed for further study of driving behavior (e.g., car following, lane changing, overtaking, etc.).

- Sight distance calculation module

The driving sight distance is one of the key factors to ensure the safety of highway driving, and it is also an important evaluation index for analyzing the highway line shape design [54]. According to Liu’s spatial sight distance measurement model with "prismatic line of sight" as the core, the DSC-BV-TI system implements the real-time calculation of driving sight distance [55]. When the user is driving virtually, the module will use the actual coordinates of the vehicle as the base point to automatically calculate the driving visual distance of the driver at the current position in real time. Moreover, it also supports data export. The sight distance information generated during driving can be exported as an Excel table file. There is a one-to-one correspondence between the sight distance and the mileage of the highway.

- Dynamic weather simulation module

This module provides a dynamic weather and time system to simulate the real environment. It can simulate weather with corresponding sound effects (e.g., sunny, cloudy, rainy, windy, snowy, foggy, thunderstorms, etc.). Much more interesting, users can use the system time or set the virtual time to realize the cycle of day and night. Based on this module, various extreme weather and light conditions can be simulated in the VR scene, and different levels of visibility can be created during driving.

- Model attributes query module

The simplified BIM model is imported into the game engine and associated with the semantic information in the BIM database based on the element ID carried by each component, so that the attribute information of the model can be queried in the VR environment. Users use the control handle to emit rays in the virtual scene. When the rays collide with the BIM model, the structure and engineering attributes will be displayed. When the design results of the BIM model are modified or adjusted, the module can synchronize the status of the BIM model to the VR system in time with the update of the BIM database.

- Traffic sign simulation module

In the DSC-BV-TI system, the simulation of traffic signs (e.g., road name plates, cordon lines, lane lines, speed limit signs, tunnel reflective rings, road signs, etc.) is realized. Users can analyze and optimize the visibility and legibility of the signs, check whether the signs indicate contradictions or are obscured by other objects, and adjust the height and position of the signs and choose the optimal sign layout to effectively navigate the driver.

- Questionnaire feedback module

This module has formulated the classification rules for the levels and categories of problems, as shown in Table 2. After viewing the space design of the transportation infrastructure, users can mark their own questions in the scene, and the information will be displayed on the mini map simultaneously. When marking in the scene, users need to specify the level and categories of the question, which is convenient for review and sorting. Ordinary users only have the authority to set "recommended" level questions, while advanced users have full authority. The questions submitted by users should include at least four items of information: person’s name, recording time, problem details and construction phase. The data in this module is stored in a database and can be exported as an Excel table file.

**Table 2:** The classification rules for the levels and categories of problems.

Classification	Details
Levels	Serious; Warning; Suggestion
Categories	Highways; Bridges; Tunnels

## 4 Application case

In order to evaluate and verify the performance of the proposed system, we implemented a case in the Jinjiazhuang Extra-Long Tunnel project of the Yan-Chong Expressway in Hebei Province, which was built for the 2022 Winter Olympics.

### 4.1 Overview of the transportation infrastructure

**Fig 4:**
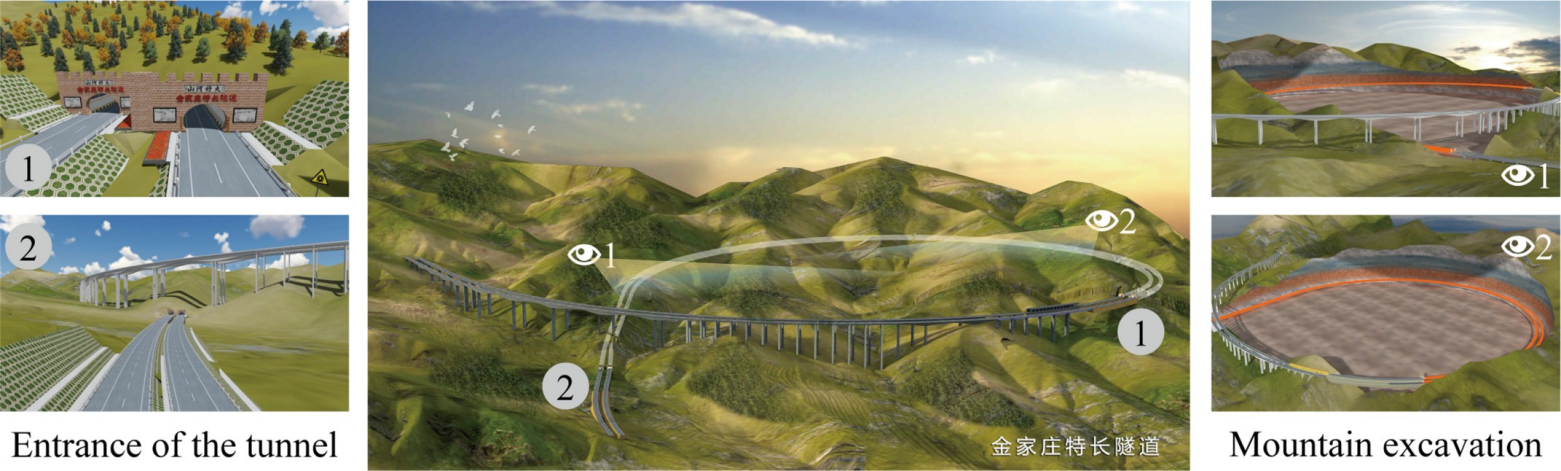
Jinjiazhuang Extra Long Tunnel.

The Jinjiazhuang Extra-Long Tunnel is an important control project for the Hebei section of the Yan-Chong (Beijing Yanqing-Zhangjiakou Chongli) Expressway for the 2022 Winter Olympics. As shown in Fig 4, it is the first extra-long spiral tunnel with a small curve radius in Hebei Province. It was recorded in the Guinness Book of World Records in August 2019 and was certified as "the longest spiral highway tunnel in the world". It is also one of the landmarks of the Yan-Chong Expressway during the Winter Olympics.

The tunnel is a two-line parallel tunnel, arranged separately for each line. The total length of the tunnel is about 4150m, the curve radius is about 860m, and it has been raised 112m from the original place through a spiral climbing design. The designers use the DSC-BV-TI system to show the planning scheme to stakeholders (i.e., acceptance experts and ordinary people), and all parties view and discuss the design results based on the DSC-BV-TI system.

**Fig 5:**
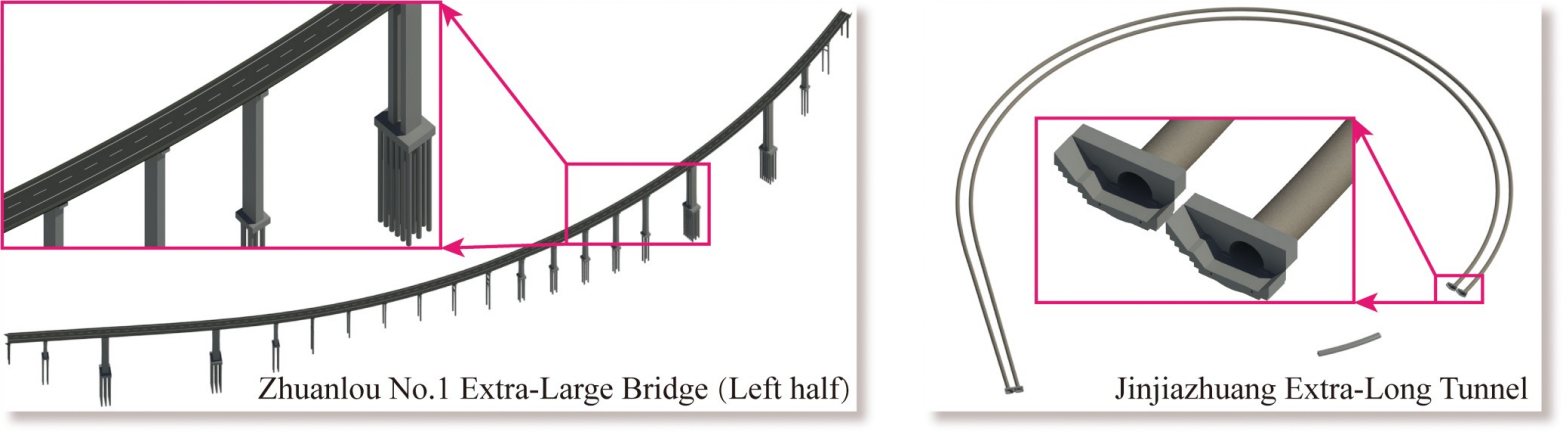
The main bridges and tunnels BIM models.

**Fig 6:**
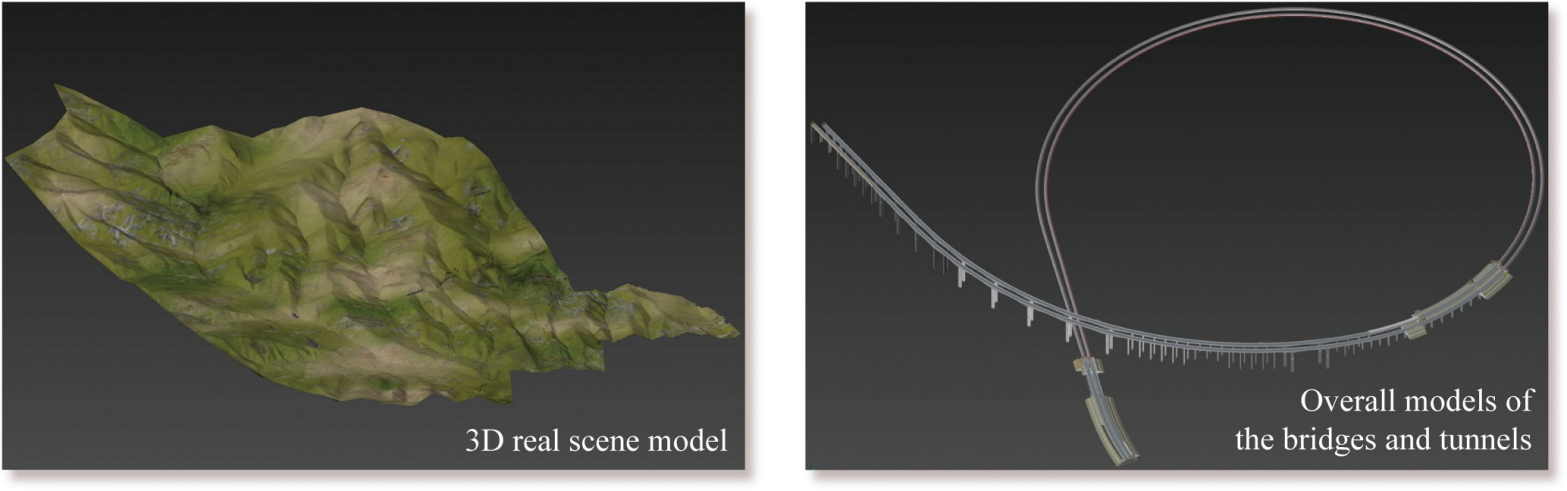
Real scene terrain and BIM models.

**Fig 7:**
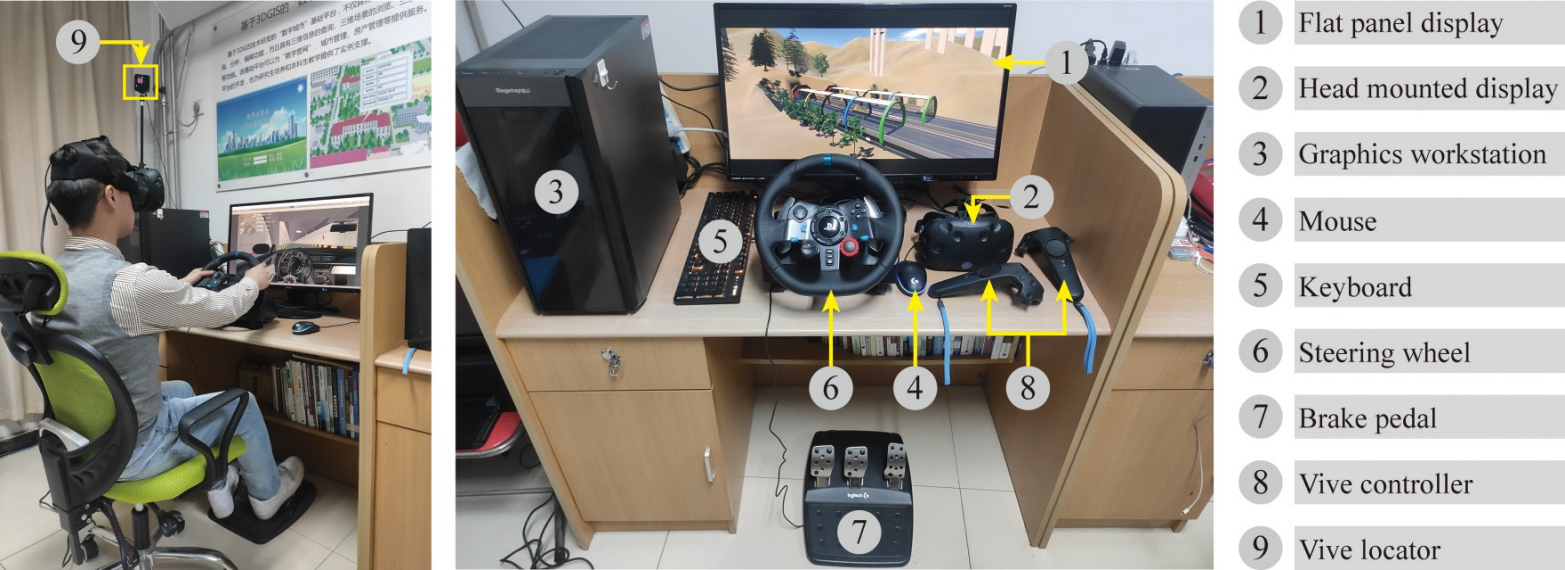
The main hardware equipment used in the case.

During the process, all the BIM models were developed for design usage. Fig 5 shows the BIM model of the main bridges and tunnels in the case study, including the left half of the Zhuanlou No.1 Extra-Large Bridge and the full-section model of the Jinjiazhuang Extra-Long Tunnel. Fig 6(A) shows the 3D real scene model based on drone tilt photography. Fig 6(B) shows the effect of the BIM model processed and rendered using 3ds Max. The system uses HTC Vive as the interactive control hardware device. The head-mounted display was used as a screen, the control handle used for function selection, and the Logitech G29 to drive the vehicle. Fig 7 shows all the hardware equipment required for system development.

### 4.2 Case realization

#### 4.2.1 Overall plan display

The Jinjiazhuang Extra-Long Tunnel is one of the landmark constructions of the Yan-Chong Expressway for the 2022 Winter Olympics, and its design should be able to show the characteristic of the Olympic Games. Inspired by the shape and color of the Olympic Rings, the design team designed the tunnel sunshade and the tunnel reflective ring, which can create good impressions and positive feelings. Based on the aerial view roaming module in the system, the space design, color planning decoration and light design of the entire transportation infrastructure have been well illustrated. The design team can show and introduce the tunnel design plan to people to help them understand the design ideas, and check whether the design results meet the requirements and people’s driving habits. Project acceptance personnel and stakeholders have obtained a complete and comprehensive understanding of the project design results through the system proposed in this article. Users can use the Vive control handle to emission a ray so as to teleport them over to the point of interest in the scene, and use the mini map to understand their location in the VR environment.

#### 4.2.2 Query the attribute information of the BIM model

**Fig 8:**
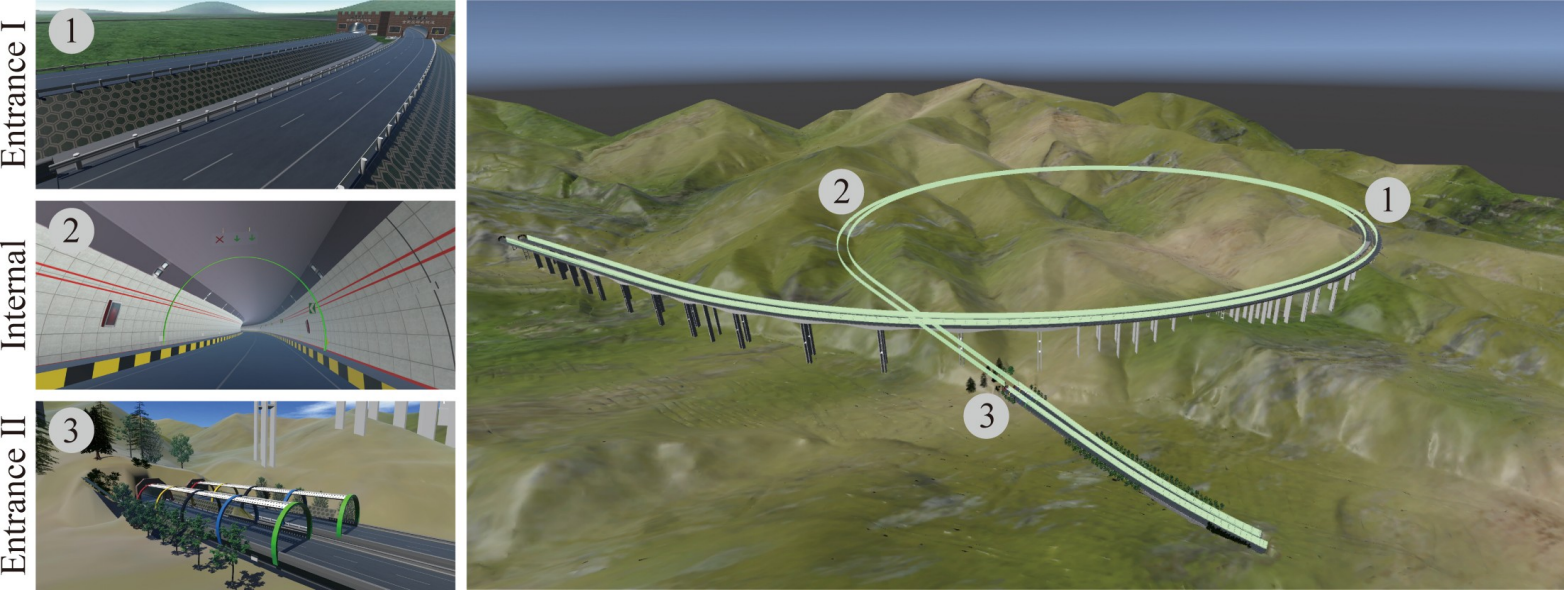
Several scenes of the DSC-BV-TI system.

The semantic information carried by the BIM model is stored in the BIM database, and the element ID is used as an index to associate with the model component in the VR environment one by one during the system development process. In the VR environment, users can use the Vive handle to emit rays as pointers to select a part of the BIM model to view information about it. With the assistance of BIM and VR integration technology, acceptance experts and other stakeholders were able to review the BIM models of traffic infrastructure such as the Zhuanlou Extra-Large Bridge and the Jinjiazhuang Extra-Long Tunnel. Fig 8 shows several scenes of the DSC-BV-TI system developed for the Jinjiazhuang Extra-Long tunnel.

#### 4.2.3 Traffic sign simulation and optimization

**Fig 9:**
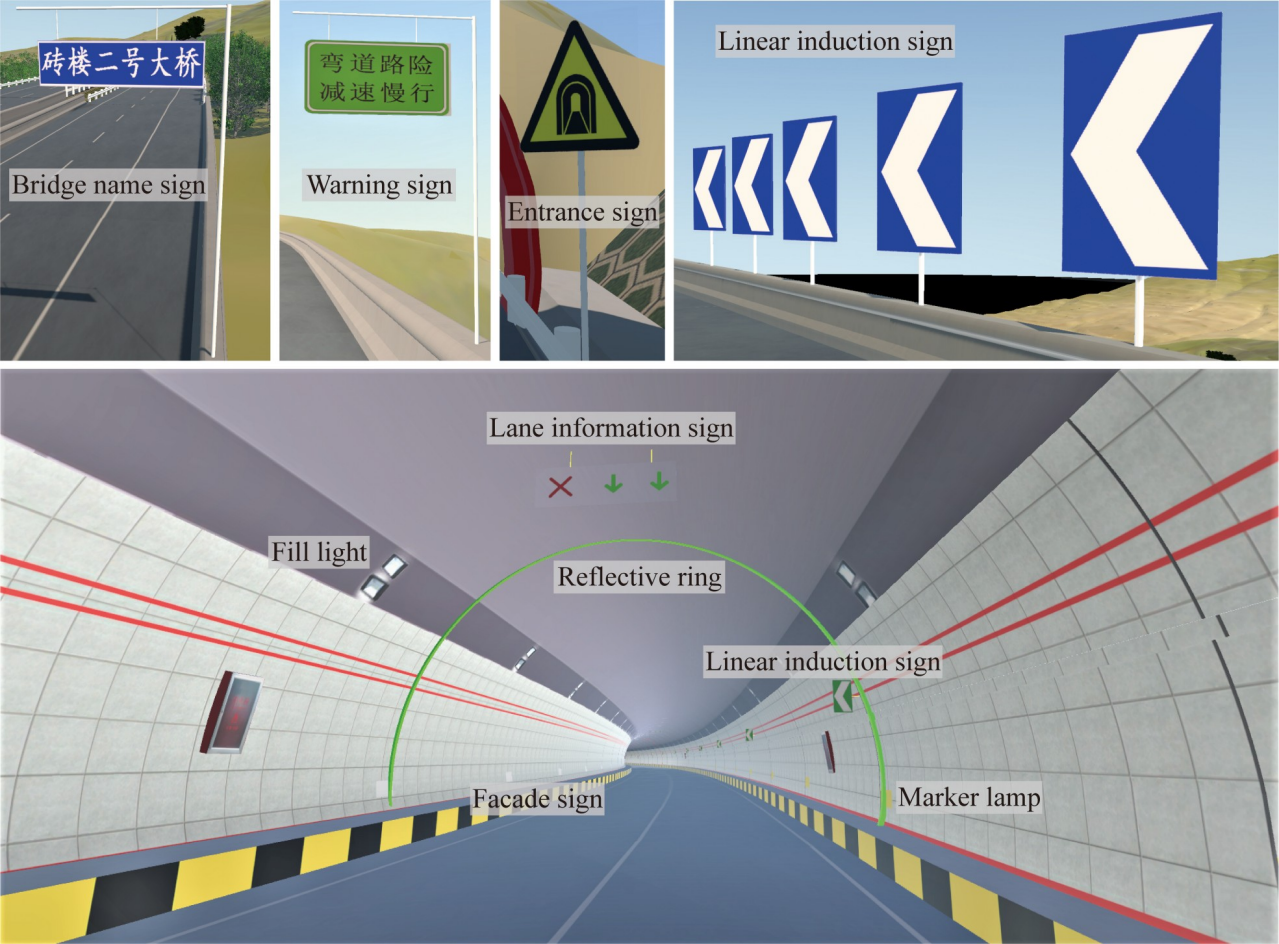
Design and simulation of traffic signs.

In the traditional method, 2D drawings are used to express the design scheme of traffic signs. Users need to have a certain amount of experience in the industry to understand the design ideas, and rely on subjective understanding to judge the rationality of the sign setting. This leads to frequent rework in the process of traffic sign design. The DSC-BV-TI system provides a way to solve this problem. After the design of the traffic sign is completed in the VR environment, relevant personnel are invited to experience and discuss the design results. Fig 9 shows the design and simulation of traffic signs.

#### 4.2.4 Virtual driving and weather simulation

**Fig 10:**
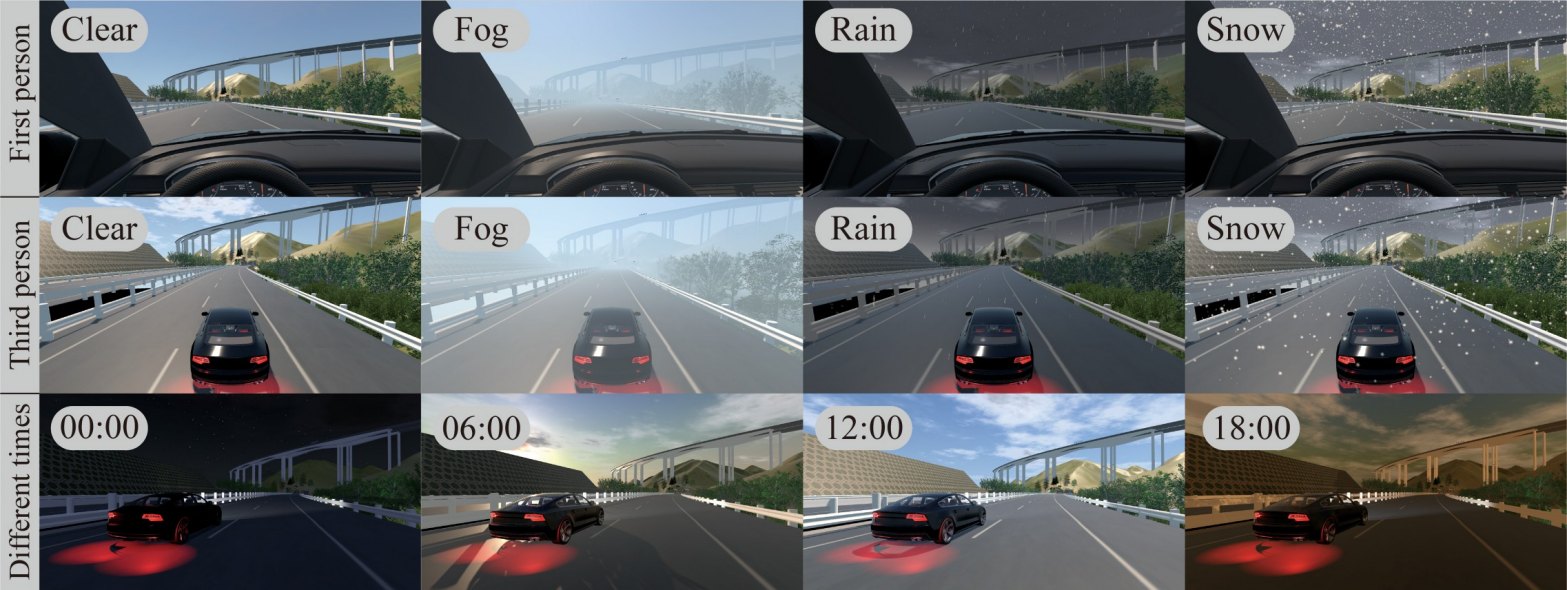
Weather and time simulation.

Using the physics engine in Unity, the virtual simulation driving of the car is realized based on vehicle dynamics. The user can use the Logitech G29 steering wheel and brake pedal to control the vehicle in the VR environment, and use the HTC Vive head-mounted display to play the driving picture. During the development of the virtual driving simulation module, the scene was rendered and light-baked, and sound effects such as accelerator, brake, and whistle were added to enhance the reality of the car driving process from the three aspects of vision, hearing and touch. This module cooperates with modules such as dynamic weather simulation that can reproduce most of the driving environment conditions in the VR environment, including various virtual scenes (e.g., sunny, foggy, rainy and snowy days, etc.). In the dynamic weather simulation module, based on Kepler’s laws of planetary motion, a real day-night cycle is realized, which can simulate physical details (e.g., light intensity and shadows, etc.) at different times. Fig 10 show the weather and time simulation in the system.

**Fig 11:**
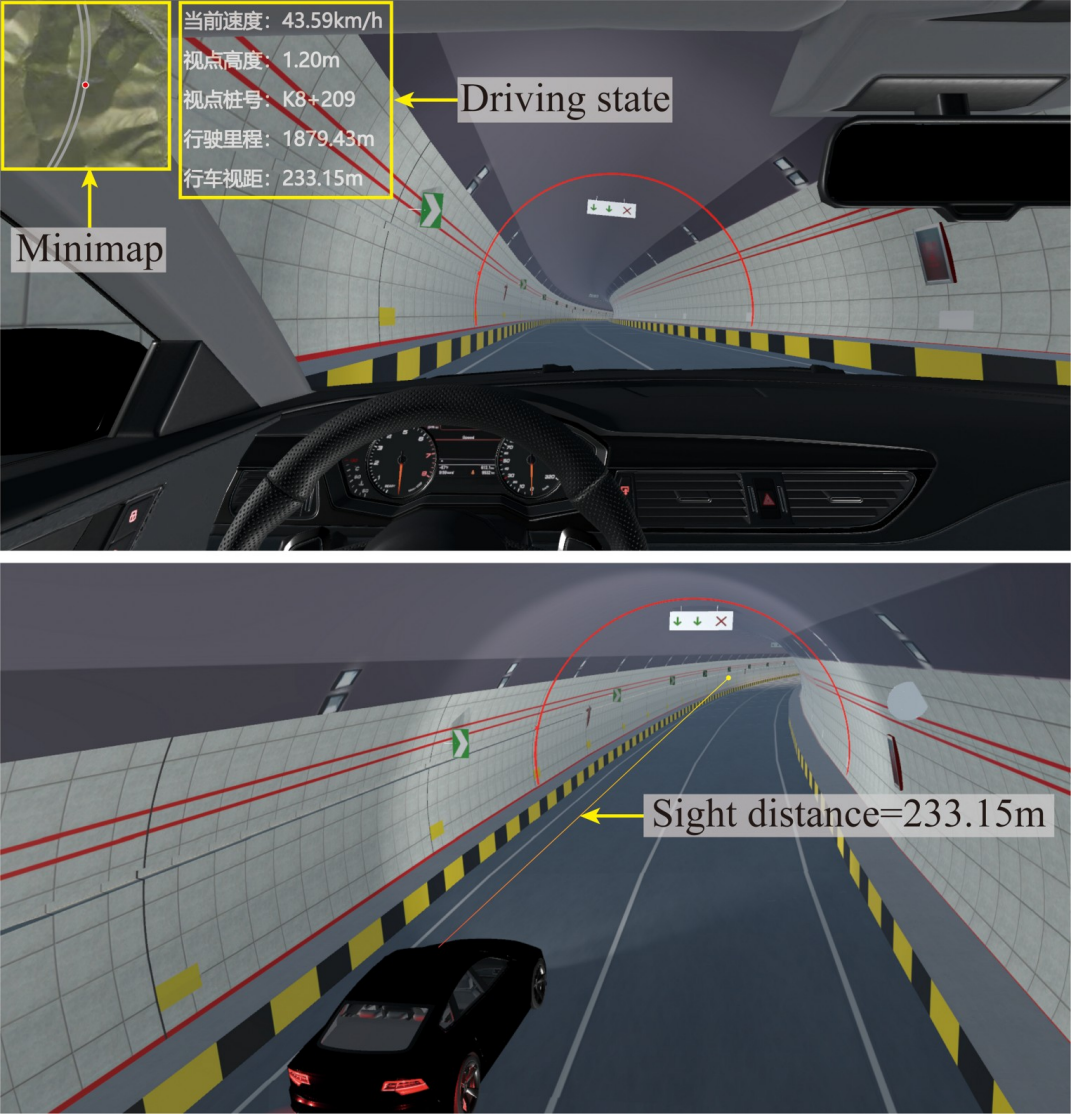
Driving state perception and sight distance calculation.

The system currently provides four types of vehicles with different performance characteristics: car, jeep, sports car and trailer. In the system, drivers can experience driving different vehicles at different times and in different weather conditions from either a first-person perspective or a third-person perspective, and review the visibility and readability of traffic signs during virtual driving. Fig 11 shows the driving state perception and real-time calculation of sight distance. This experience is difficult to achieve in the traditional CAD/BIM-based 2D/3D design method. Traditional methods cannot reproduce realistic driving scenes, and the actual user experience of the road cannot be known in advance before the project is complete.

#### 4.2.5 Driving state perception and sight distance calculation

When driving, the current speed, viewpoint height, viewpoint station number, mileage, and driving sight distance of the vehicle can be obtained in real time. These data have been stored in the system database in a sequence of time intervals and can be exported as an Excel table file. In this project, the original maximum design speed in the Jinjiazhuang Extra-Long Tunnel is 80km/h, which meets the national standards for tunnel speed limits. However, in the actual driving experience, it was found that the long continuous curve in the tunnel may easily cause visual fatigue during driving and increase the hidden danger of traffic accidents. After discussion, the maximum design speed of the tunnel was limited to 60km/h. This incident shows that the DSC-BV-TI system can play a key role in the design process of transportation infrastructure.

#### 4.2.6 Questionnaire collection and feedback

Using the DSC-BV-TI system, the design team can modify the sign design before customizing the traffic signs based on the questionnaire feedback. The corrected BIM model will be updated in the system to enhance the design quality of traffic signs and save costs. In addition, users can discover potential safety hazards in the design of bridges, tunnels, highways, etc. through virtual simulation driving in the system.

The design team designed BIM models of bridges, tunnels and expressways based on engineering requirements and standard specifications, and applied them to the DSC-BV-TI system. After reviewing the BIM model, the inspector can use the question collection and feedback module to put forward their own review opinions on the buildings in the VR scene (for example, it is recommended to set up the tunnel entrance sign farther away from the tunnel entrance). Subsequently, the building will be simultaneously marked on the mini map, and the corresponding markings will be distinguished by different colors (red, yellow, green) according to the different opinion levels (serious, warning, suggestion).

In this module, the SQL Server database is used to record all the details of the problem, including the person who proposed it, the time of recording, the profession, the details of the problem, the suggestion, etc. All the information can be used for discussion and analysis in the DSC-BV-TI system. In the end, the designer modifies the BIM model of the project again based on the users’ feedback, and the user can review the modified design results again through the system. Therefore, the inspectors can perform a simulation review and analysis in the system, collect and organize various problems through the questionnaire feedback module, and give effective feedback on the proposed ideas to the design team, thereby helping to optimize the engineering design results.

In order to verify the effectiveness of using the system for design communication, a questionnaire was designed to investigate the opinions of users and the design team. All participants were invited to fill in the questionnaire after using the system, and their answers were recorded in the DSC-BV-TI system in an anonymous or non-anonymous form (according to the user’s preference), and the design team performed statistics and analysis on them.

### 4.3 Case verification

#### 4.3.1 Experimental method

In order to test the role of the DSC-BV-TI system in verifying the design plan in the transportation infrastructure project, we built a special game scene for the Jinjiazhuang Extra-Long Tunnel. The aim of the game is to use the DSC-BV-TI system, in a sunny and dry experimental scene, each participant relies on his own driving habits to drive a car from a position 1km away from the entrance of the tunnel until it passes through the entire tunnel, stopping at the 1km position after the tunnel exit. One experiment on each lane will be conducted on the left and right lanes of the tunnel.

We invited 28 drivers from the stakeholders of the project to conduct the experiment: Table 3 shows a summary of the basic information of the participants in the experiment. They all have a full driving license and no driving simulation syndrome. Each driver will be informed of the operating specifications to be followed, and will be proficient in the operating skills of simulated driving in the DSC-BV-TI system. All their behavior during the game will be recorded and used as a basis for optimizing the tunnel design after the experiment.

**Table 3:** General information of drivers.

Statistical parameter	Average (years)	Range (years)	Standard deviation (years)
Age	36.7	28~42	3.1
Years of driving experience	8.7	4~15	3.2

#### 4.3.2 Experimental conclusions

According to the experimental results of the virtual driving simulation in the BV system, the following are the key conclusions drawn in relation to the optimization of tunnel design schemes:

1) The signs for driving lights in the tunnel should be supported by supplementary textual signs.

**Fig 12:**
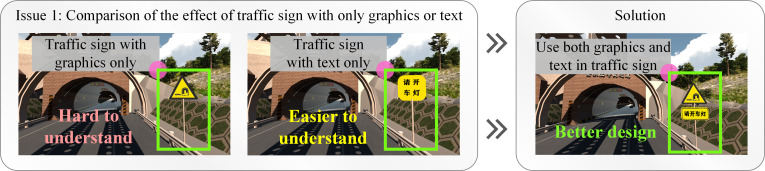
The design optimization of signs for driving lights.

Turning on the lights in the tunnel is of great significance for improving traffic safety. Through the experiment, more than 75% of the interviewees believed that, compared to the graphic “tunnel driving lights warning signs”, plain text prompts could help them understand the meaning of the signs more quickly. In the end, the design team adopted a combination of graphic signs and supplementary textual signs to strengthen the recognition of the signs. Fig 12 shows the design optimization of signs for driving lights in the tunnel.

2) The location of traffic signs should avoid drivers’ scotopic/photopic adaptation stage to ensure effective visual distance.

**Fig 13:**
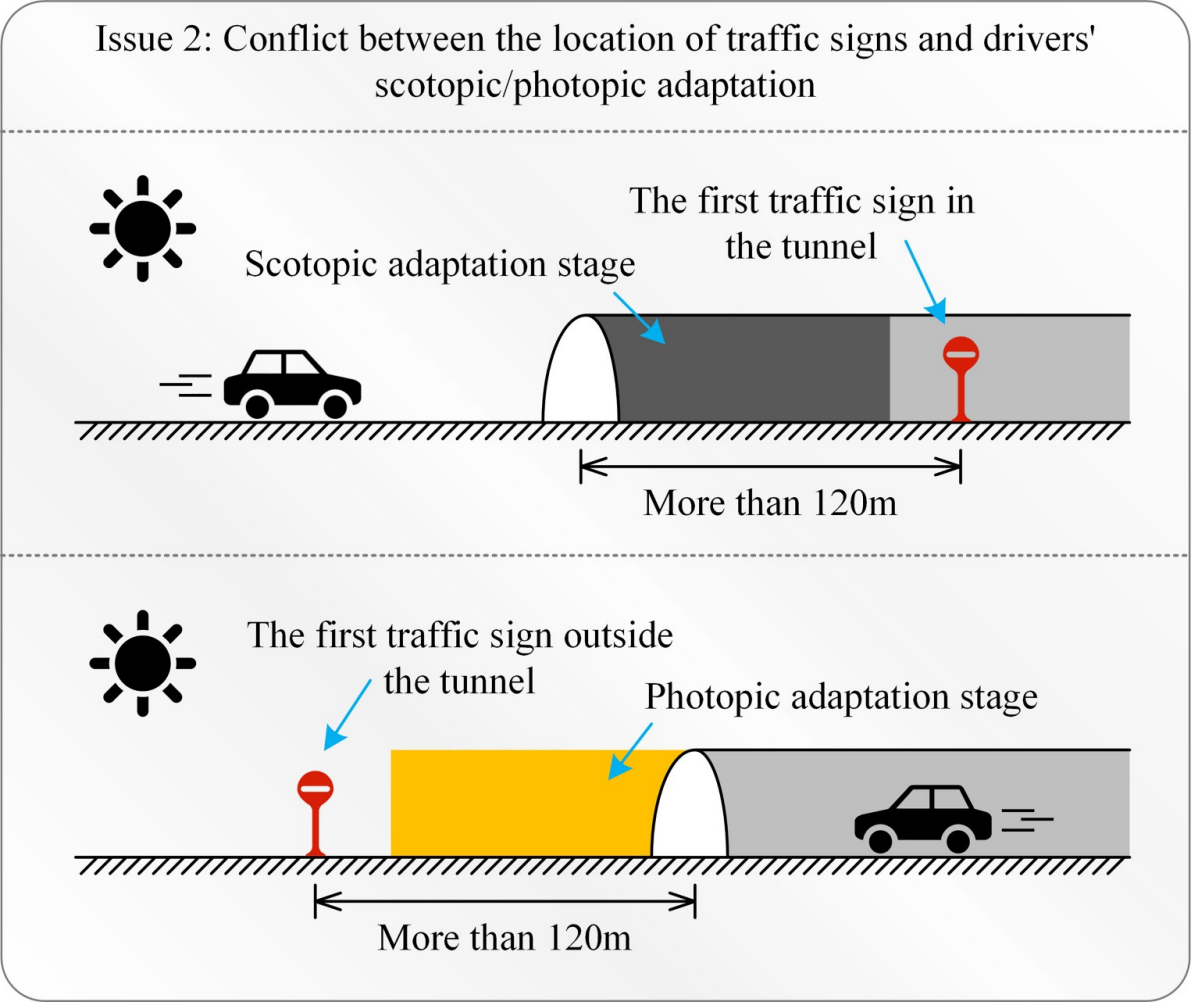
Scotopic and photopic adaptation.

When entering or exiting the tunnel, due to the difference in the intensity of light inside and outside the tunnel, the driver will be temporarily blinded. Therefore, to ensure that drivers can see the signs clearly, this period of visual blindness should be avoided. To this end, according to the corresponding relationship between the visual recognition distance of traffic signs and the size of the signs specified by China, combined with the actual experience of the driver, the distance between the first traffic sign in the tunnel and the tunnel entrance, or the first traffic sign outside the tunnel and the end of the tunnel were re-examined and adjusted. The distance between the outlets is specified to be greater than 120m. Fig 13 shows the location of traffic signs and drivers’ scotopic/photopic adaptation.

3) The tunnel exit distance warning sign should be set up together with a speed limit sign.

According to the simulated driving experiment results carried out using the DSC-BV-TI system, the tunnel exit distance warning sign may prompt drivers to expect to leave the tunnel as soon as possible, thereby increasing the speed of the vehicle. Therefore, the design team puts the tunnel exit distance warning sign and a speed limit sign together in the tunnel.

4) Speed limit signs should be set repeatedly in the tunnel.

**Fig 14:**
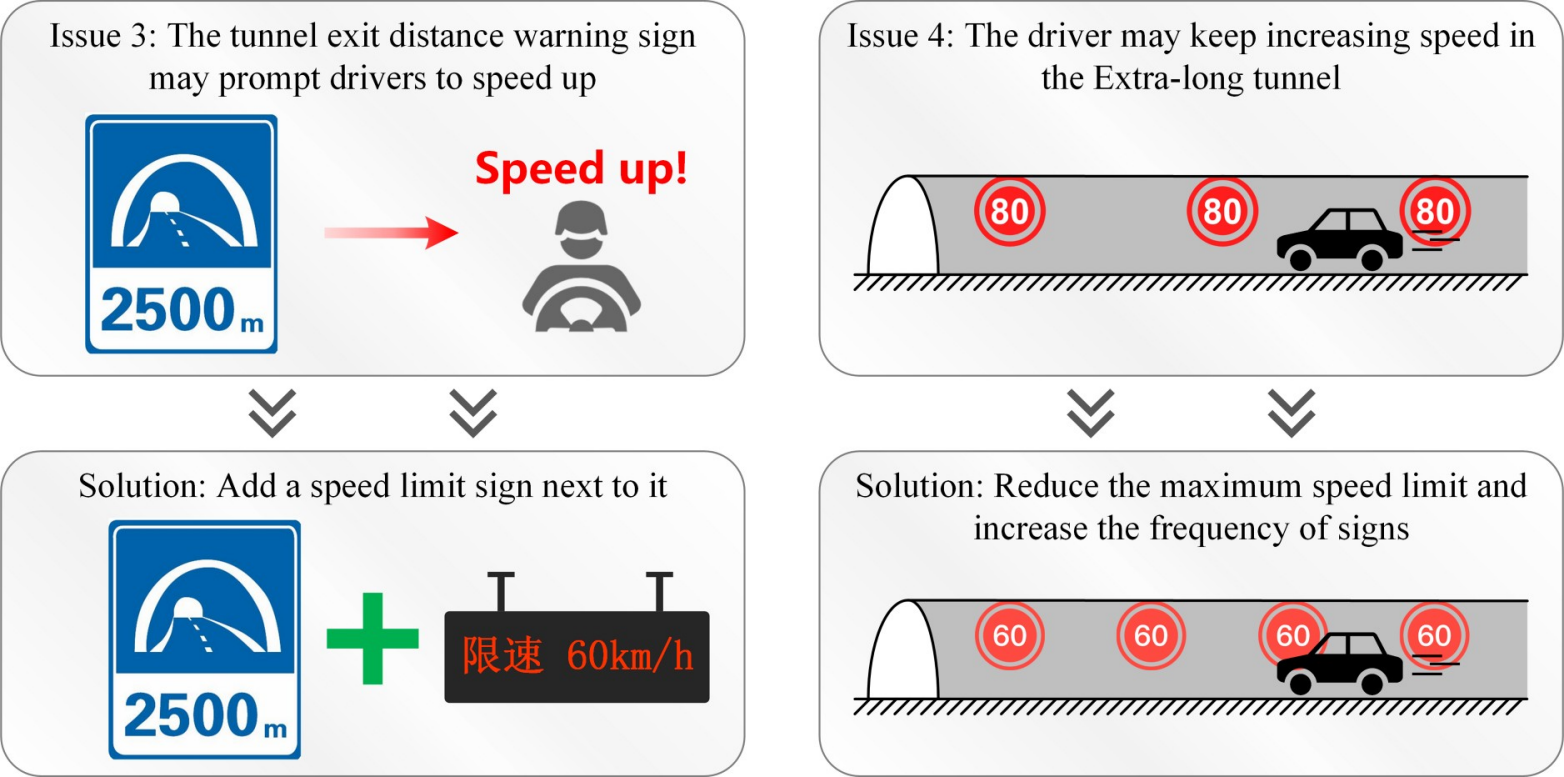
Other optimizations.

According to the statistical results of the vehicle speed, it was found that, due to the long-term impact of the closed, monotonous and repetitive driving environment in the tunnel, the driver will experience psychological changes and the speed of the car will increase in stages, especially in the continuous line of the tunnel. On downhill sections, speeding is more likely to occur. After discussion, the speed limit in the tunnel was reduced from the original 80km/h to 60km/h, and the frequency of speed limit plates was increased to ensure safe driving in the tunnel. Fig 14 shows some other optimizations of the speed of cars in the tunnel and traffic signs.

### 4.4 Case application analysis and discussion

#### 4.4.1 Application overview

It took almost 3 months to develop and test the simulation system based on Unity. Of this time, it took 3 weeks to establish and optimize the BIM model in the system. The system took about 6 weeks from prototype design to development, and 3 weeks for invited designers and non-designers to discuss the system, analysis and design results, and to optimize collaboration. Fortunately, Unity’s handy component-based development and abundant plug-in resources considerably shortened the development cycle of the system and saved considerable economic and labor costs.

We investigated the performance of the 8 main functional modules of the DSC-BV-TI system. During the investigation, users were asked to score the 8 modules of the system according to their subjective feelings (20 to 100, with an interval of 10. They were not allowed to award the same score for multiple modules). Finally, the average of the scores of each module was calculated.

**Fig 15:**
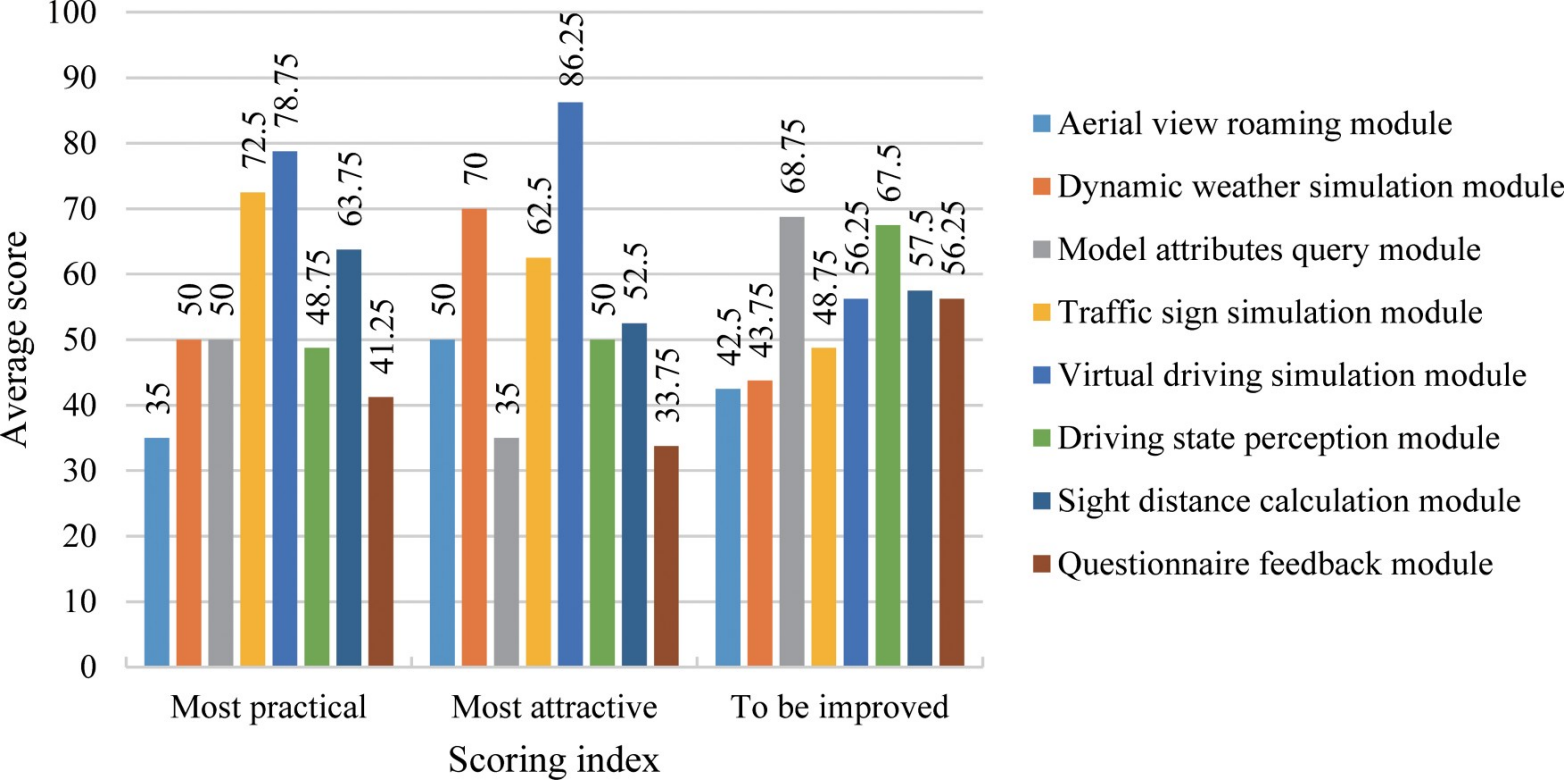
Survey result of modules’ performance.

Fig 15 shows the results of all users’ investigation of the performance of the 8 modules in the system. The virtual driving simulation module, the traffic sign simulation module, and the sight distance calculation module were considered the three most practical modules by users. We found that these three are functions that cannot be achieved in the traditional CAD/BIM-based 2D/3D design methods, but they played an indispensable role in the linear design of expressways, traffic sign design and speed limit design.

In addition, users believed that the virtual driving simulation module, dynamic weather simulation module and traffic sign simulation module were the three most attractive modules in the DSC-BV-TI system. The comparison results show that building a realistic simulated roaming environment and a human-computer interaction system that conforms to usage habits can help enhance users’ willingness to use the system.

Finally, we picked modules with inadequate performance in order to optimize the system in depth in the future. The results show that the model attribute query module and the driving state perception module were considered to be two modules which needed to be improved. Users said that querying BIM model attributes in the VR environment was cumbersome and meaningless, not to mention that this goal can also be achieved through BIM software. As for the driving state perception module, users thought that the amount of information obtained was small. If it were possible to calculate real-time behavior variables, such as the time headway and distance headway between vehicles while driving, it can conveniently provide driving behavior data for subsequent research work.

#### 4.4.2 Advantages

The DSC-BV-TI system integrates BIM, VR and game engine development technology, providing designers, acceptance experts and stakeholders with immersive roaming, design inspection and driving simulation. Based on the results of the case study, the main advantages of the system are summarized as follows:

1. Compared with traditional CAD/BIM-based 2D/3D design methods, the DSC-BV-TI system can provide project participants with a realistic three-dimensional visual expression during the design stage of transportation infrastructure, and realize the simulation of the design.
2. The questionnaire feedback module is integrated in the DSC-BV-TI system, which means it can efficiently collect and sort review comments into logical order, save communication time, and speed up the overall design process of the project.
3. The DSC-BV-TI system integrates BIM and VR technologies. According to the results of the case study, the simulation effect is better than using the BIM model alone.
4. During the virtual driving simulation, the DSC-BV-TI system can display status information of the vehicle, such as speed, mileage, etc., and can also calculate the driver’s visual distance in real time at the current location. The sight distance information can be exported to an Excel worksheet for statistics and analysis according to mileage. Compared with similar racing games, the function is very unique and useful in the sight distance analysis of spiral tunnels.
5. After the user uses the system to perform a simulation check on the project, the feedback information, such as comments and suggestions put forward will be stored in the SQL Server database and can be exported in batches for designers to view and modify the model. This module provides convenience for remote interaction between the inspectors and the designers.
6. In the DSC-BV-TI system, a head-mounted display can be used to display the driving scene, and a steering wheel combination which can simulate real force feedback can be used to control the vehicle.
7. Based on Unity’s excellent rendering capabilities and physics engine, the system can realistically express the design ideas, and it can also conveniently compare and select design schemes according to different weather conditions.
8. Using the traffic sign simulation module of the DSC-BV-TI system, users can view the form, placement and angle of a sign and its visibility under different weather conditions, thereby optimizing the design of traffic signs.

#### 4.4.3 Limitations

Some shortcomings and limitations of the DSC-BV-TI system were also discovered during the application of the case:

1. During the system development process, although mature plug-in and component development was used to reduce the difficulty of research and development, save costs and cycles, compared to the traditional way, developing the system does add extra work.
2. Wearing the head-mounted display for a long time can easily cause the user to have VR motion sickness. In virtual driving, the motion sickness will befall more quickly due to the faster vehicle speed. Although a flat-panel display can be used as an alternative, it will reduce the immersive experience of the scene accordingly. However, an advanced virtual reality display with a higher screen refresh rate can be used to alleviate this problem to a certain extent.
3. The adaptation of the BIM model is still a major time-consuming and energy-consuming process, and the design team may need to modify the model several times based on feedback.
4. The virtual driving module of the system currently only supports single-person operation and single-vehicle driving, and cannot really simulate traffic flow. In the future, it is planned to build a web-based server on this basis and store system data in the cloud to realize multi-vehicle collaborative driving.

#### 4.4.4 Suggestions

Finally, based on the understanding in the research, the following suggestions are put forward:

1. The purpose of developing the system is to allow users to obtain an ideal simulation experience, thereby creating an efficient design discussion environment. In case the virtual simulation system is difficult to use or deviates too much from the real world, it will hinder communication between all parties instead. Therefore, when using advanced information technology to design a virtual simulation system, it is necessary to ensure that it has good usability, particularly in the construction of terrain and ground object models in order to conform to the real effect.
2. During the integration process of BIM and VR, a series of optimizations should be carried out to ensure that the system runs smoothly. Due to construction needs, the BIM model is very delicate, which also leads to the model file being too large. When integrating the model as a scene element, it is necessary to reduce the weight, such as deleting unnecessary faces, simplifying the grid, merging repeated objects, etc., and selectively rendering elements in the camera’s field of view to reduce the burden of the computer.
3. The cost and cycle of developing the system is a factor that cannot be ignored. Therefore, it is recommended to use mature and fully functional plug-ins in the development process to enhance the efficiency and stability of system development.

## 5 Conclusions

In the transportation infrastructure design project, communication difficulties caused by differences in professional knowledge levels between the design team and other stakeholders are the main problems faced in the collaborative work process. This research takes the Jinjiazhuang Extra-Long Tunnel as a case, integrates BIM and VR technology in the game engine, and develops the DSC-BV-TI system. It allows users to review BIM models in an immersive VR environment, reduces communication barriers, helps designers, investors, and the government choose the best implementation plan, and can provide demonstration and reference for related projects.

The DSC-BV-TI system proposed by this study has 4 characteristics: First, in view of the particularity of the transportation infrastructure, the HTC Vive virtual reality device and the Logitech G29 steering wheel were used as hardware devices, so that the simulation and driving experience could be replicated to a high level. Second, the combination of various modules can simulate different road conditions and light/dark conditions, which helps to optimize the design results of traffic signs. Third, the system innovatively integrates the real-time sight distance calculation and driving state sensing modules. These two modules played a role in determining the tunnel speed limit. Fourth, users can mark their questions and opinions on the corresponding construction elements during the review process, and the marked information will be displayed on the mini map simultaneously. The designers can use the system to automatically sort and summarize the problems, and finally modify the design results based on the feedback.

According to the questionnaire survey results of users using the DSC-BV-TI system, the DSC-BV-TI system can provide a better visual communication effect than the traditional CAD/BIM-based 2D/3D design, and enhance the user’s perception and understanding of the project. On the basis of virtual driving simulation, the DSC-BV-TI system integrates modules such as dynamically changing weather, driving status and sight distance, so that the designers can optimize the design of traffic signs and tunnel speed limits. The users’ critical comments and suggestions can be collected, sorted and exported by using the database, which can save time and economic costs. Finally, based on the analysis of the advantages and disadvantages of the DSC-BV-TI system, suggestions for improving the system are given from the perspectives of user experience, development process and R&D cost. The results of the case study show that, in the future design of transportation infrastructure, the use of the DSC-BV-TI system proposed by this research can promote the realization of the IPD mode and help stakeholders to obtain better design solutions.

In view of the characteristics of transportation infrastructure, using a BIM model combined with VR technology to form a closed-loop engineering design and optimize framework in the design stage, which has a propelling effect on the realization of the IPD mode in the transportation infrastructure field.

There are two main directions for future research: First, on the basis of the existing functions of the DSC-BV-TI system, it can be used in the operation and maintenance stage of the project to realize the full life cycle management of the transportation infrastructure. Second, further discussion can take place on how to realize multi-user collaborative communication in the DSC-BV-TI system, so plans can be developed to simulate more complex traffic conditions, such as traffic flow.
